# Transcriptomic Analysis of *Listeria monocytogenes* in Response to Bile Under Aerobic and Anaerobic Conditions

**DOI:** 10.3389/fmicb.2021.754748

**Published:** 2021-11-11

**Authors:** Damayanti Chakravarty, Gyan Sahukhal, Mark Arick, Morgan L. Davis, Janet R. Donaldson

**Affiliations:** ^1^Cell and Molecular Biology, The University of Southern Mississippi, Hattiesburg, MS, United States; ^2^Institute for Genomics, Biocomputing & Biotechnology, Mississippi State University, Mississippi State, MS, United States; ^3^Department of Biological Sciences, Mississippi State University, Mississippi State, MS, United States

**Keywords:** *Listeria monocytogenes*, transcriptomics, anaerobiosis, bile, stress response, anaerobic

## Abstract

*Listeria monocytogenes* is a gram-positive facultative anaerobic bacterium that causes the foodborne illness listeriosis. The pathogenesis of this bacterium depends on its survival in anaerobic, acidic, and bile conditions encountered throughout the gastrointestinal (GI) tract. This transcriptomics study was conducted to analyze the differences in transcript levels produced under conditions mimicking the GI tract. Changes in transcript levels were analyzed using RNA isolated from *L. monocytogenes* strain F2365 at both aerobic and anaerobic conditions, upon exposure to 0 and 1% bile at acidic and neutral pH. Transcripts corresponding to genes responsible for pathogenesis, cell wall associated proteins, DNA repair, transcription factors, and stress responses had variations in levels under the conditions tested. Upon exposure to anaerobiosis in acidic conditions, there were variations in the transcript levels for the virulence factors internalins, listeriolysin O, etc., as well as many histidine sensory kinases. These data indicate that the response to anaerobiosis differentially influences the transcription of several genes related to the survival of *L. monocytogenes* under acidic and bile conditions. Though further research is needed to decipher the role of oxygen in pathogenesis of *L. monocytogenes*, these data provide comprehensive information on how this pathogen responds to the GI tract.

## Introduction

*Listeria monocytogenes* is a gram-positive foodborne pathogen that is responsible for the disease listeriosis (Scallan et al., 2011). Pregnant women, infants, elderly, and immunocompromised individuals are more susceptible to listeriosis, with meningitis, septicemia, and spontaneous abortions being possible manifestations of the disease (Thigpen et al., 2011). Being a foodborne pathogen, this bacterium must be able to respond to the stressors encountered following ingestion of contaminated food. Low pH, bile, and hypoxic/anoxic environments are some of the key stressors that are encountered by *L. monocytogenes* within the gastrointestinal (GI) tract (Davis et al., 1996).

Low pH of the stomach is one of the initial stressors encountered by *L. monocytogenes* upon ingestion (White et al., 2015). The low pH of the gastric secretion is a roadblock to invasion by the bacteria. *Listeria’s* acid response involves the SOS response, LisRK (a two-component regulatory system that regulates listerial osmotolerance), components of sigma B regulon, ATPase proton pump, and enzymatic systems that regulate internal hydrogen ion concentration (Sleator and Hill, 2005). A transcriptomic study that was performed on *Listeria* grown in the presence of organic acids revealed an increase in the transcript levels of sigma B and *prfA* regulated genes, which included internalins, phospholipases, and other virulence genes. This previous study also indicated an up-regulation of oxidative stress defenses, DNA repair, intermediary metabolism, cell wall modification, and cofactor and fatty acid biosynthesis (Tessema et al., 2012). A proteomic study performed on *Listeria* grown in the presence of organic salts demonstrated an up-regulation of oxidoreductases and lipoproteins. Upon exposure to hydrochloric acid, it was also observed that proteins involved in respiration (enzyme dehydrogenases and reductases), osmolyte transport, protein folding and repair, general stress resistance, flagella synthesis and metabolism were expressed in the response to the acidic conditions (Bowman et al., 2012).

*Listeria* is also exposed to bile within the GI tract (White et al., 2015). Bile is synthesized by the liver and stored in the gall bladder. It is released into the duodenum during digestion (Monte et al., 2009). The bile acids are the antibacterial component of bile; bile acids induce damage to the cell wall and DNA (Coleman et al., 1979; Bernstein et al., 1999; Prieto et al., 2004, 2006). Within the gall bladder, bile is found at a nearly neutral pH (7.5), while in the duodenum it is more acidic (pH 5.5) (White et al., 2015). Bile is more bactericidal at acidic pH than at a neutral pH, as indicated in a study that showed a decrease in survival in bile under pH 5.5 in comparison to a pH of 7.5 (Dowd et al., 2011). Many studies have been conducted to determine the global response of *L. monocytogenes* to bile encountered within the GI tract. For instance, the transcription factor *brtA*, which senses cholic acid and regulates efflux pumps (MdrM and MdrT) is involved in bile tolerance (Quillin et al., 2011). Bile salt hydrolases neutralize conjugated bile acids, thereby providing protection against the bactericidal properties of bile (Dowd et al., 2011). The *bilE* gene is also involved in detoxifying bile acids (Dowd et al., 2011).

In addition to changes in pH and bile, *L. monocytogenes* is also exposed to changes in oxygen concentrations. The duodenum is considered microaerophilic in nature, while the gall bladder is anaerobic (Zheng et al., 2015). Oxygen availability has been found to influence bile resistance. A proteomics study performed under anaerobic conditions in the presence of bile observed notable alterations in cell wall associated proteins, DNA repair proteins and oxidative stress response proteins. Under anaerobic conditions the *Listeria* adhesion protein has been observed to have a significant role in intestinal infection (Burkholder et al., 2009). Additionally, oxygen deprivation has been found to affect the survival of *L. monocytogenes in vitro* (Payne et al., 2013; Wright et al., 2016), as well as in cell cultures, guinea pigs (Bo Andersen et al., 2007), and gerbils (Harris et al., 2019). These studies highlight the importance of oxygen in regulation of virulence. However, it is not known what the transcriptomic response of *L. monocytogenes* is to conditions that mimic the GI tract under physiologically relevant anaerobic conditions. Therefore, the goal of this study was to determine the impact of oxygen on the transcriptomic response of *L. monocytogenes* to bile in conditions that mimic the duodenum (pH 5.5) and the gall bladder (pH 7.5).

## Results

### Survival of *L. monocytogenes* in Conditions Mimicking Gastrointestinal Tract

**FIGURE 1:**
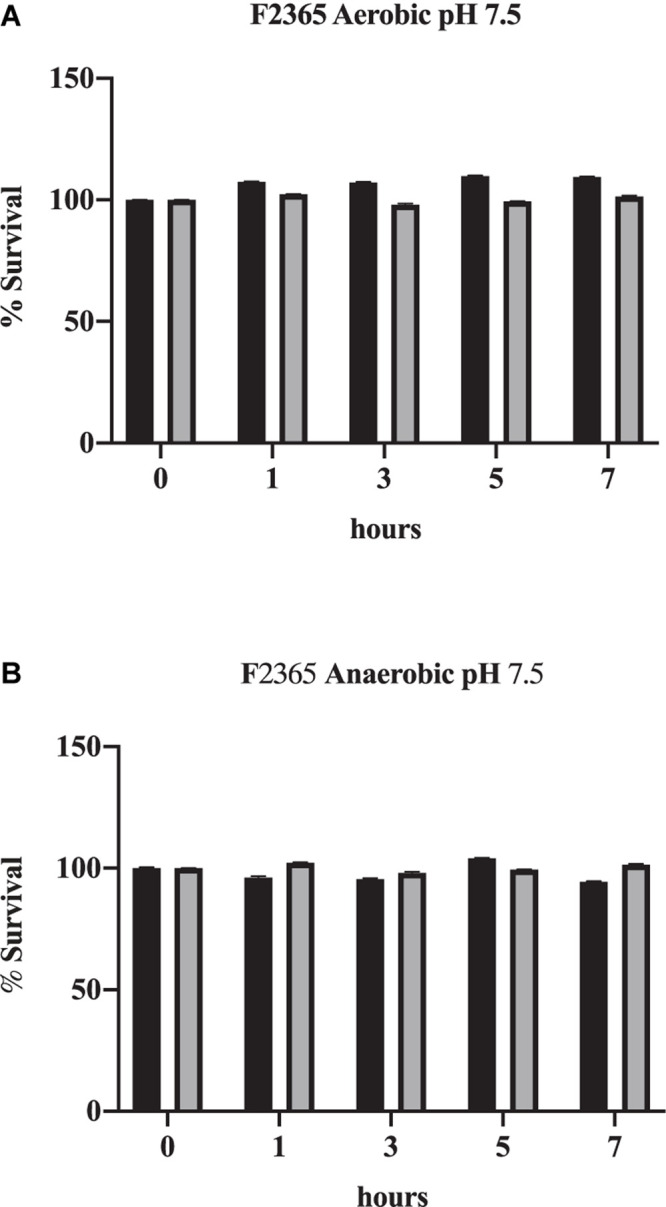
Impact of oxygen on the survival of *L. monocytogenes* in conditions mimicking the gall bladder. F2365 was exposed to either aerobic **(A)** or anaerobic **(B)** conditions with 0% bile (black bars) or 1% bile (gray bars) at a pH of 7.5 and survival was measured by viable plate counts over 7 h. Data represent averages of three independent replicates. Error bars represent standard deviation from biological replicates.

*Listeria monocytogenes* exhibits slightly slower growth rates under anaerobic conditions (Figures 1A vs. 1B). Bile also impacted the viability of *L. monocytogenes* strain F2365 differently under anaerobic conditions. Under neutral pH, bile did not have a significant impact on survival of *L. monocytogenes* strain F2365 under either aerobic (Figure 1A) or anaerobic conditions (Figure 1B).

**FIGURE 2:**
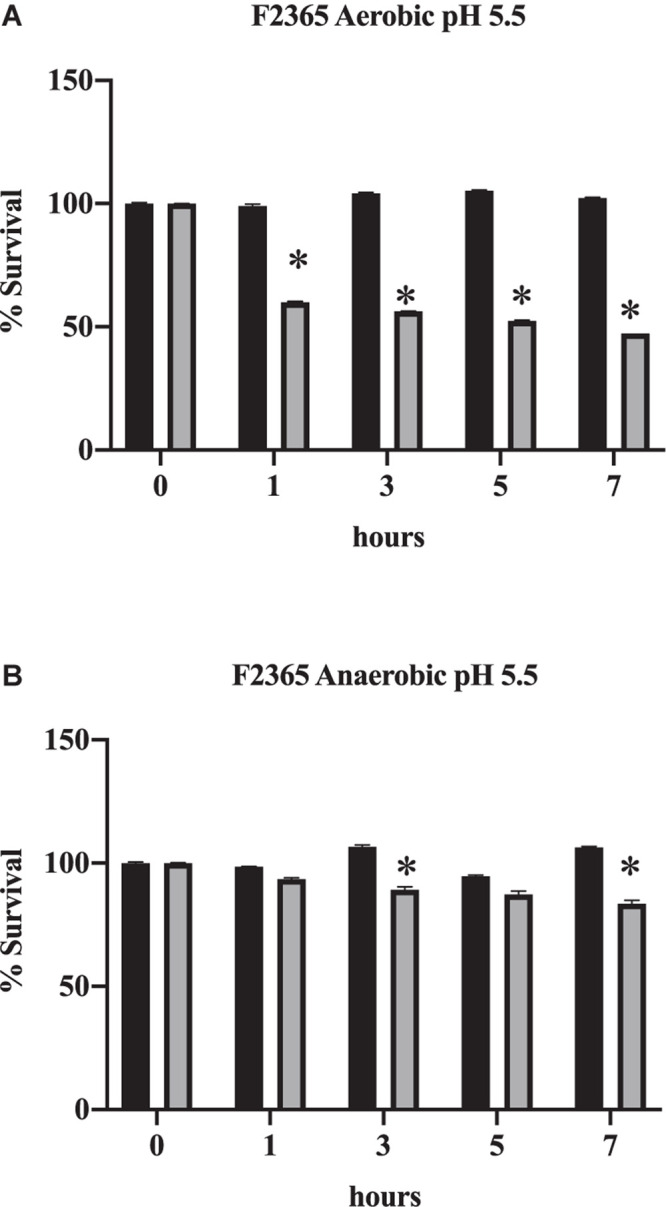
Impact of oxygen on the survival of *L. monocytogenes* in conditions mimicking the duodenum. F2365 was exposed to either aerobic **(A)** or anaerobic conditions **(B)** with 0% bile (black bars) or 1% bile (gray bars) at a pH of 5.5 and survival was measured by viable plate counts over 7 h. Data represent averages of three independent replicates. Error bars represent standard deviation from biological replicates. * Indicates *p* < 0.05 in comparison to time 0.

At acidic pH in the presence of bile, which mimics the exposure to bile in the duodenum, the percentage of *L. monocytogenes* that survived significantly declined (Figure 2A; *p* < 0.05). This further demonstrates the increase in toxicity exhibited by bile when in acidic conditions. Survival also declined under anaerobic conditions in comparison to time 0 h (Figure 2B, *p* < 0.05). However, the decrease in viability was not as severe under anaerobic conditions (Figure 2B) in comparison to aerobic conditions (Figure 2A; *p* < 0.05). This indicates that anaerobic conditions improve the survival of *L. monocytogenes* to the toxic effects of bile.

### Overall Changes in Transcript Levels in Response to Conditions Mimicking the Gastrointestinal Tract

As significant alterations in survival were observed following 1 h of bile exposure under acidic conditions, this time point was selected to compare the impact that oxygen had on the transcriptome. Table 1 shows the overall changes in transcripts detected. Under anaerobic conditions, a total of 190 transcripts in media at pH 7.5 and 268 at pH of 5.5 were identified to be differentially expressed in comparison to aerobic conditions. In the presence of bile and absence of oxygen, 304 and 434 transcripts were differentially produced at pH 7.5 and 5.5, respectively. Under anaerobic conditions, upon exposure to bile, variations in the transcript levels of 200 genes were identified at pH 7.5 and 419 at pH 5.5. For all conditions tested, there were globally more transcripts identified to be up-regulated than down-regulated, except for acidic bile conditions under anaerobic growth.

**TABLE 1:** Total changes in transcript levels following exposure to bile at pH of 7.5 or 5.5 under either aerobic or anaerobic conditions.

	**Aerobic vs. Anaerobic**	**Bile Aerobic vs. Bile Anaerobic**	**Anaerobic vs. Bile Anaerobic**
pH 7.5	Total = 190	Total = 304	Total = 200
	Up = 125 Down = 65	Up = 207 Down = 97	Up = 131 Down = 69
pH 5.5	Total = 268	Total = 434	Total = 419
	Up = 147 Down = 121	Up = 213 Down = 221	Up = 264 Down = 155

### Changes in Transcript Levels in Response to Anaerobic Conditions

Transcripts representative of five genes were found to be increased in expression levels under exposure to anaerobic conditions regardless of whether the cultivation was conducted under either neutral or acidic pH (Table 2 and Supplementary Figure 1). These included genes involved in membrane transport, protein folding, and stress response. Of these transcripts the amino acid transporter (*LMOf2365_2333*) had nearly a 9-fold increase in levels at neutral pH in comparison to acidic pH. Transcripts representative of the *dnaJ* (*LMOf2365_1491*) and *dnaK* (*LMOf2365_1492*) genes, which encode for molecular chaperones and have roles in phagocytosis and protein homeostasis, were also increased under anaerobic conditions at both pH conditions tested. The transcript representative of the *cadA* (*LMOf2365_0672*) gene, which encodes for a heavy metal translocating P-type ATPase and is a component of the CadAC efflux cassette, was also increased 6.1-fold at pH 7.5 and 3.8 at pH 5.5 under oxygen depleted conditions (Table 2).

**TABLE 2:** Transcript levels increased in response to anaerobiosis at pH 7.5 or 5.5.

**Gene ID**	**Gene product**	**Transcript fold changes**	
	***Membrane transport***	***pH 7.5***	***pH 5.5***
*cadA LMOf2365_0672*	Cadmium translocating P-type ATPase	6.1	3.8
*LMOf2365_2333*	Amino acid antiporter	137.2	15.0
***Protein folding***			
*dnaK LMOf2365_1492*	Chaperone protein	5.3	8.6
*dnaJ LMOf2365_1491*	Chaperone protein	7.2	4.8
***Stress response***			
*gadG LMOf2365_2405*	Glutamate decarboxylase gamma	11.2	3.2

The transcript levels of 18 genes were decreased under anaerobic conditions regardless of the pH condition tested (Table 3 and Supplementary Figure 2). Out of these eighteen transcripts, six were representative of uncharacterized hypothetical proteins; all of these had lower transcript levels under neutral conditions in comparison to acidic conditions. This could suggest that these hypothetical genes are regulated similarly. The remaining transcripts identified encoded for stress response, membrane associated protein, and metabolism protein (Table 3).

**TABLE 3:** Transcript levels decreased in response to anaerobiosis at pH 7.5 or 5.5.

**Gene ID**	**Gene product**	**Transcript fold changes**	
		**pH 7.5**	**pH 5.5**
***Membrane transport***			
*LMOf2365_2554*	Sensor histidine kinase	−4.46	−12.1
***Metabolism***			
*acpP LMOf2365_1834* *LMOf2365_0511*	Acyl carrier protein Heme oxygenase (staphylobilin-producing)	−13.6	−5.3
*gcvT LMOf2365_1365*	Glycine cleavage system T protein	−8.9	−4.7
*LMOf2365_0585*	Phosphoglycerate mutase family protein	−7.7	−3.6
***Stress response***			
*LMOf2365_0544*	Universal stress protein family	−5.9	−5.8
***Hypothetical proteins***			
*LMOf2365_0964*	Conserved hypothetical protein	−13.7	−5.9
*LMOf2365_0511*	Conserved hypothetical protein	−13.6	−5.3
*LMOf2365_1087*	Conserved hypothetical protein	−12.1	−3.9
*LMOf2365_0808*	Conserved hypothetical protein	−11.2	−3.1
*LMOf2365_1179*	Hypothetical protein	−8.3	−3.7
*LMOf2365_2288*	Conserved hypothetical protein	−6.3	−5.8

### Changes in Transcript Levels in Response to Anaerobic Acidic Conditions

In acidic conditions, transcript levels of 140 genes were increased (Table 4 and Supplementary Figure 1) and 104 were decreased under anaerobiosis (Table 5). Analyzing these transcripts up-regulated in response to acidic conditions under anaerobiosis revealed that several biological pathways related to pathogenesis, stress response, membrane associated proteins, transcription factors and DNA repair mechanisms influenced the survival of *L. monocytogenes* (Table 4). Transcripts representative of genes involved in metabolism, transcription factor and pathogenesis were down-regulated (Table 5). Certain transcripts encoding for glycolytic enzymes increased under acidic anaerobic conditions as well (Table 4). These included the glyceraldehyde-3-phosphate dehydrogenase (5.4-fold increase), phosphoglycerate mutase (4.7-fold increase), and pyruvate kinase (6.7-fold increase).

**TABLE 4:** Transcript levels increased for select genes in response to anaerobiosis at pH 5.5.

**Gene ID**	**Gene product**	**Transcript fold changes**
***Metabolism***		
*hemL LMOf2365_1574*	Glutamate-1-semialdehyde-2,1-aminomutase	3.1
*nrdD LMOf2365_0299*	Anaerobic ribonucleoside-triphosphate reductase	3.1
*LMOf2365_1386*	Phosphate acetyl/butyryltransferase family protein	3.1
*panD LMOf2365_1929*	Aspartate 1-decarboxylase	3.1
*LMOf2365_0434*	Polysaccharide deacetylase family protein	3.1
*pepQ LMOf2365_1600*	Proline dipeptidase	3.1
*ldh-2 LMOf2365_1553*	L-lactate dehydrogenase	3.2
*LMOf2365_2670*	N-acetylmuramoyl-L-alanine amidase, family 4	3.3
*LMOf2365_1275*	Hydrolase, alpha/beta fold family	3.3
*LMOf2365_0372*	Transcriptional regulator, DeoR family	3.4
*LMOf2365_2200*	Putative lactoylglutathione lyase	3.4
*LMOf2365_0846*	Pyruvate flavodoxin/ferredoxin oxidoreductase	3.4
*LMOf2365_0277*	Glycosyl hydrolase, family 1	3.7
*asnB LMOf2365_1687*	Asparagine synthase (glutamine-hydrolyzing)	3.8
*pfl-1 LMOf2365_1425*	Formate acetyltransferase	3.8
*LMOf2365_2673*	Orn/Lys/Arg decarboxylase	3.9
*LMOf2365_0330*	Threonine aldolase family protein	4.1
*mvaS LMOf2365_1434*	Hydroxymethylglutaryl-CoA synthase	4.2
*LMOf2365_1633*	Putative glutamyl-aminopeptidase	4.3
*LMOf2365_1642*	Dipeptidase	4.3
*LMOf2365_0603*	Glycosyl hydrolase, family 1	4.4
*LMOf2365_0550*	Glycosyl hydrolase, family 4	4.6
*pnp LMOf2365_134*	Polyribonucleotide nucleotidyltransferase	4.6
*Gpm LMOf2365_2238*	Phosphoglycerate mutase	4.7
*LMOf2365_1226*	Putative peptidase	5.2
*LMOf2365_2528*	Putative fructose-bisphosphate aldolase	5.3
*gap LMOf2365_2432*	Glyceraldehyde-3-phosphate dehydrogenase, type I	5.4
*LMOf2365_1083*	Inositol monophosphatase family protein	5.5
*LMOf2365_2199*	Metallo-beta-lactamase family protein	5.6
*LMOf2365_1400*	Putative acylphosphatase	5.7
*LMOf2365_1299*	4-hydroxybenzoyl-CoA thioesterase family protein	6.2
*Pyk LMOf2365_1592*	Pyruvate kinase	6.7
*ldh-1 LMOf2365_0221*	L-lactate dehydrogenase	7.5
*pflA LMOf2365_1426*	Pyruvate formate-lyase activating enzyme	7.6
*galU LMOf2365_1099*	UTP-glucose-1-phosphate uridylyltransferase	7.7
*LMOf2365_0582*	CBS domain protein	8.5
*LMOf2365_2144*	Nitroreductase family protein	9.3
*LMOf2365_0802*	Putative acyl-carrier protein phosphodiesterase	9.4
*ald LMOf2365_1601*	Alanine dehydrogenase	11.9
*manA LMOf2365_2143*	Mannose-6-phosphate isomerase, class I	13.6
*LMOf2365_1608*	Putative inorganic polyphosphate/ATP-NAD kinase	13.6
*LMOf2365_2308*	Aminopeptidase C	13.9
*pfl-2 LMOf2365_1946*	Formate acetyltransferase	40.3
*murI LMOf2365_1246*	Glutamate racemase	68
***Transcription factors***		
*LMOf2365_2140*	Transcriptional regulator, DeoR family	3.1
*argR LMOf2365_1384*	Arginine repressor	3.2
*LMOf2365_1526*	DNA-binding response regulator	4.1
*LMOf2365_1907*	Iron-dependent repressor family protein	4.3
*LMOf2365_0755*	Transcriptional regulator, PadR family	4.6
*LMOf2365_0480*	Putative transcriptional regulator	4.8
*LMOf2365_1986*	Transcriptional regulator, Fur family	4.8
*LMOf2365_0814*	Transcriptional regulator, MarR family	7.8
*LMOf2365_1707*	Peroxide operon transcriptional regulator	8.6
***Pathogenesis***		
*LMOf2365_1812*	Internalin family protein	5.4
*hly LMOf2365_0213*	Listeriolysin O	10.2
***Motility***		
*LMOf2365_1723*	Methyl-accepting chemotaxis protein	4.4
***DNA repair***		
*topA LMOf2365_1293*	DNA topoisomerase I	3.3
*nth LMOf2365_1923*	Endonuclease III	3.5
*exoA LMOf2365_1807*	Exodeoxyribonuclease	4.2
*LMOf2365_1643*	MutT/nudix family protein	4.4
*ung-2 LMOf2365_1236*	Uracil-DNA glycosylase	5.3
***Stress response***		
*LMOf2365_1997*	Putative tellurite resistance protein	3.1
*LMOf2365_0783*	Glyoxalase family protein	3.4
*LMOf2365_0963*	Peroxide resistance protein Dpr	3.5
*LMOf2365_2735*	General stress protein 26	5.1
*LMOf2365_1121*	Glyoxalase family protein	5.2
***Protein folding***		
*groEL LMOf2365_2099*	Chaperone protein GroEL	4.0
*atpB LMOf2365_2508*	ATP synthase F0, A subunit	4.1

**TABLE 5:** Transcript levels decreased for select genes in response to anaerobiosis at pH 5.5.

**Gene ID**	**Gene product**	**Transcript fold changes**
***Metabolism***		
*pheA LMOf2365_1555*	Prephenate dehydratase	−18.8
*LMOf2365_2263*	Putative arsenate reductase	−14.8
*LMOf2365_1556*	GTP-binding protein, GTP1/OBG family	−13.4
*LMOf2365_0148*	Ser/Thr protein phosphatase family protein	−13.2
*LMOf2365_2831*	Sucrose phosphorylase	−9.3
*LMOf2365_0128*	Lipase	−8.9
*cah LMOf2365_0827*	Carbonic anhydrase	−8.9
*LMOf2365_2647*	Galactitol PTS system EIIA component	−8.5
*tkt-3 LMOf2365_2640*	Transketolase	−6.2
*arcA LMOf2365_0052*	Arginine deiminase	−6.1
*LMOf2365_2643*	Alcohol dehydrogenase, zinc-dependent	−5.7
*qoxA LMOf2365_0016*	Cytochrome aa3-600 menaquinol oxidase subunit II, Oxidative phosphorylation	−5.5
*gabD LMOf2365_0935*	Succinate-semialdehyde dehydrogenase	−5.4
*LMOf2365_2364*	Ferredoxin/flavodoxin—NADP+ reductase	−5.3
*LMOf2365_0209*	UDP-N-acetylglucosamine pyrophosphorylase	−4.9
*guaB LMOf2365_2746*	Inosine-5′-monophosphate dehydrogenase	−4.3
*LMOf2365_0566*	Putative N-carbamoyl-L-amino acid amidohydrolase	−4.1
*ctaB LMOf2365_2088*	Heme o synthase	−4.1
*prs-1 LMOf2365_0210*	Ribose-phosphate pyrophosphokinase	−3.9
*LMOf2365_1048*	Metallo-beta-lactamase family protein	−3.6
*LMOf2365_2576*	Acetamidase/formamidase family protein	−3.4
*LMOf2365_2824*	Glycosyl transferase, family 65	−3.0
***Transcription Factors***		
*ada, LMOf2365_0093*	AraC family transcriptional regulator	−9.4
*LMOf2365_0127*	Transcriptional regulator, AraC family	−7.2
*purr LMOf2365_0203*	Pur operon transcriptional repressor	−4.3
*LMOf2365_1683*	Phosphosugar-binding transcriptional regulator, RpiR family	−4.2
*LMOf2365_0023*	Transcriptional regulator, GntR family	−4.0
*LMOf2365_2467*	Phosphate transport system protein PhoU	−4.0
*LMOf2365_2017*	LacI family transcriptional regulator	−3.3
*LMOf2365_2224*	ArsC family protein, regulatory protein spx	−3.3
*LMOf2365_1010*	Transcriptional regulator, MarR family	−3.1
***Membrane Transport***		
*LMOf2365_1428*	MFS transporter, ACDE family, multidrug resistance protein	−7.9
*LMOf2365_2542*	Peptide/nickel transport system substrate-binding protein; bacterial extracellular solute-binding protein, family 5	−7.7
*LMOf2365_2575*	Putative Mg2+ transporter-C (MgtC) family protein	−5.4
*LMOf2365_0759*	Methyl-accepting chemotaxis protein	−4.2
*LMOf2365_0267*	Sugar ABC transporter, sugar-binding protein	−4.0
*LMOf2365_0167*	Peptide/nickel transport system substrate-binding protein	−3.9
*LMOf2365_2351*	Multicomponent Na+ :H+ antiporter subunit A	−3.3
*LMOf2365_0876*	Sugar ABC transporter, sugar-binding protein	−3.1
*LMOf2365_2732*	ATP-binding cassette, subfamily B, bacterial AbcA/BmrA	−3.1
***Pathogenesis***		
*LMOf2365_0128*	Lipase	−8.9
*inlE LMOf2365_0283*	Internalin E	−6.7
*LMOf2365_2467*	Phosphate transport system protein PhoU	−4.0

### Changes in Transcript Levels in Response to Bile Under Anaerobic Conditions

Transcripts representative of 53 genes were found to be up-regulated in response to exposure to bile under anaerobic conditions (Table 6 and Supplementary Figure 3). Transcripts encoding for transcription regulators of virulence, antibiotic resistance, metabolism, and membrane associated proteins were also observed to increase in their levels of expression (Table 6). Transcripts representative of nine genes were down-regulated under anaerobic conditions in presence of bile at both pH 7.5 and 5.5 (Table 7 and Supplementary Figure 4). Fold changes of the transcript levels of genes associated with metabolism, translation, pathogenesis, and transcription were down-regulated (Table 7).

**TABLE 6:** Transcript levels increased for select genes in response to anaerobiosis at pH of 7.5 and 5.5.

**Gene ID**	**Gene product**	**Transcript fold changes**	
	***Transcription factors***	***pH 7.5***	***pH 5.5***
*LMOf2365_0641*	Transcriptional regulator, MarR family	6.5	13.7
*prfA LMOf2365_0211*	Listeriolysin regulatory protein	11.5	3.7
*LMOf2365_1986*	Fur family transcriptional regulator, ferric uptake regulator	12.7	18.8
*glnR LMOf2365_1316*	Transcriptional repressor GlnR	13.6	13.9
***Metabolism***			
*LMOf2365_2358*	Thioesterase family protein	4.2	6.4
*LMOf2365_0884*	ATP-dependent RNA helicase DeaD	4.4	3.1
*LMOf2365_1433*	Acetyl-CoA acetyltransferase	4.5	6.6
*LMOf2365_1729*	Deoxynucleoside kinase family protein	4.6	10.5
*LMOf2365_1660*	Muramoyltetrapeptide carboxypeptidase	5.1	4.4
*cysK LMOf2365_0234*	Cysteine synthase A	6.1	6.2
*LMOf2365_1038*	Putative PTS system, glucose-specific, IIA component	6.3	4.4
*LMOf2365_2371*	NifU family protein	6.9	27.1
*Cah LMOf2365_0827*	Carbonic anhydrase	7.1	7.2
*LMOf2365_1419*	Acetyltransferase, GNAT family	7.3	3.7
*trxB LMOf2365_2451*	Selenocompound metabolism	8.7	5.0
*glnA LMOf2365_1317*	Glutamine synthetase, type I	9.9	3.3
*LMOf2365_2364*	Pyridine nucleotide-disulfide oxidoreductase family protein	10.1	5.1
*LMOf2365_0861*	Putative endoribonuclease L-PSP	10.6	4.2
*LMOf2365_0391*	Messenger RNA biogenesis	10.7	7.8
*divIVA LMOf2365_2045*	Cell division protein DivIVA	14.1	5.1
*LMOf2365_0997*	Acetyltransferase, GNAT family	14.5	7.1
*alsS LMOf2365_2030*	Acetolactate synthase	16.5	20.8
*LMOf2365_0640*	Flavodoxin-like fold domain protein	35.9	37.4
***Membrane transport***			
*LMOf2365_0761*	Putative membrane protein	4.0	6.0
*LMOf2365_2229*	Oligopeptide ABC transporter, oligopeptide-binding protein	4.3	3.6
*LMOf2365_1443*	Transporter, NRAMP family	5.7	6.3
*LMOf2365_0168*	Zinc ABC transporter, zinc-binding protein	6.9	52.5
*LMOf2365_1435*	Putative transporter	8.2	7.4
*LMOf2365_1012*	Membrane protein, TerC family	9.6	257.7
*LMOf2365_2330*	Putative membrane protein	18.9	46.3

**TABLE 7:** Transcript levels decreased for select genes in response to anaerobiosis at pH 7.5 and 5.5.

**Gene ID**	**Gene product**	**Transcript fold changes**	
		**pH 7.5**	**pH 5.5**
***Metabolism***			
*adhE LMOf2365_1656*	Acetaldehyde dehydrogenase/alcohol dehydrogenase	−48.1	−71.2
*LMOf2365_0250*	Serine O-acetyltransferase	−5.8	−4.4
*murE LMOf2365_2070*	UDP-N-acetylmuramoyl-L-alanyl-D-glutamate–2,6-diaminopimelate ligase	−5.7	−4.5
***Translation***			
*LMOf2365_2879*	tRNA-Glu	−25.3	−4.8
*LMOf2365_2913*	tRNA Leu	−11.5	−4.1
*hly LMOf2365_0213*	Listeriolysin O	−70.0	−3.7
***Transcription factors***			
*LMOf2365_2205*	Sigma-54 dependent transcriptional regulator	−10.7	−5.5

### Changes in Transcript Levels in Response to Bile Under Acidic and Anaerobic Conditions

Transcript levels of 210 genes were up-regulated in response to bile at acidic pH under anaerobic conditions (Table 8 and Supplementary Figure 3). Transcripts encoding for transcription factors, metabolism, replication and repair, cell signaling, protein folding, and pathogenesis were also found to be up-regulated. Additionally, transcripts representing 146 genes were down-regulated under anaerobic conditions with acidic bile (Table 9 and Supplementary Figure 4), with these being primarily associated with metabolism, membrane transport, replication and repair, pathogenesis, and transcription factors.

**TABLE 8:** Transcript levels increased for select genes in response to bile in anaerobic conditions at pH 5.5.

**Gene ID**	**Gene product**	**Transcript levels**
***Metabolism***		
*LMOf2365_0638*	Rhodanese-like domain protein	3.4
*LMOf2365_0686*	Serine/threonine protein phosphatase family protein	4.1
*mvaS LMOf2365_1434*	Hydroxymethylglutaryl-CoA synthase	4.8
*LMOf2365_1406*	Putative pyrroline-5-carboxylate reductase	38
***Pathogenesis***		
*inlE LMOf2365_0283*	Internalin E	3.6
*LMOf2365_0508*	Putative antigen	4.4
*LMOf2365_2725*	CBS domain protein	5.2
*hlY-III LMOf2365_1893*	Hemolysin III	6.2
*LMOf2365_0726*	Flagellin	29.2
*LMOf2365_1503*	DNA-binding protein, ComEA family	130.5
***Cell Signaling***		
*LMOf2365_0626*	Cyclic nucleotide-binding protein	6.8
***Protein Folding***		
*LMOf2365_1018*	ATP-dependent Clp protease, ATP-binding subunitE	3.9
*clpP LMOf2365_2441*	ATP-dependent Clp protease, protease subunit	5.2
*trx-1 LMOf2365_1242*	Thioredoxin	6.2
*clpP-1 LMOf2365_1146*	ATP-dependent Clp protease, proteolytic subunit P	25.0
***Membrane Transport***		
*LMOf2365_0153*	Oligopeptide ABC transporter	3.0
*LMOf2365_0288*	Putative transporter	3.1
*LMOf2365_2265*	CBS domain protein	3.1
*LMOf2365_0295*	Competence protein ComEC/Rec2-related protein	3.3
*LMOf2365_1088*	Cell division protein, FtsW/RodA/SpoVE family	3.3
*LMOf2365_1219*	Putative membrane protein	3.4
*acsA LMOf2365_2700*	Acetyl-coenzyme A synthetase	3.6
*LMOf2365_2554*	Sensor histidine kinase	3.7
*LMOf2365_2835*	Major facilitator family transporter	3.7
*LMOf2365_2647*	PTS system, IIA component	3.8
*zurM-2 LMOf2365_1465*	Zinc ABC transporter, permease protein	4.0
*LMOf2365_0622*	Formate/nitrite transporter family protein	4.0
*LMOf2365_1002*	Drug resistance transporter, EmrB/QacA family	4.7
*LMOf2365_0930*	Putative membrane protein	5.0
*LMOf2365_0967*	Putative transporter	5.1
*LMOf2365_0810*	Putative membrane protein	5.6
*LMOf2365_1721*	Cation efflux family protein	6.4
*LMOf2365_0588*	Magnesium transporter, CorA family	6.5
*LMOf2365_0701*	ABC transporter, ATP-binding protein	7.1
*lmrB-2 LMOf2365_2560*	Lincomycin resistance protein LmrB	7.3
*LMOf2365_1695*	Putative laminin-binding surface protein	8.2
*LMOf2365_2119*	MATE efflux family protein	8.5
*LMOf2365_2222*	CoiA-like family protein	10.6
*LMOf2365_0570*	ABC transporter, substrate-binding protein	12.0
*LMOf2365_0812*	RarD protein	13.6
*LMOf2365_0941*	ABC transporter, ATP-binding protein	18.1
*LMOf2365_1011*	MATE efflux family protein	19.1
*LMOf2365_0167*	Bacterial extracellular solute-binding protein	20.4
*LMOf2365_1502*	Zinc-binding, ComEB family protein	21.8
*LMOf2365_1428*	Major facilitator family transporter	25.6
*LMOf2365_1000*	ABC transporter, ATP-binding protein	46.6
*LMOf2365_0034*	Putative membrane protein	60.2
***Replication and Repair***		
*LMOf2365_0196*	Deoxyribonuclease, TatD family	3.1
*LMOf2365_1533*	ATPase, AAA family domain protein	3.3
*LMOf2365_1998*	Putative DNA-damage-inducible protein P	4.2
*LMOf2365_0949*	Putative DNA-3-methyladenine glycosylase	4.7
*rnhA LMOf2365_1909*	Ribonuclease HI	4.9
*LMOf2365_2784*	Replication and repair	5.9
*dbpA LMOf2365_1260*	ATP-dependent RNA helicase DbpA	8.4
*recA LMOf2365_1417*	Recombination protein RecA	9.2
*LMOf2365_0863*	Excinuclease ABC subunit C domain protein	11.4
*LMOf2365_2339*	MutT/nudix family protein	11.6
*LMOf2365_0849*	Putative transposase OrfA, IS3 family	12.7
*dnaG LMOf2365_1474*	DNA primase	18.7
***Transcription Factors***		
*LMOf2365_1427*	Transcriptional regulator, PadR family	3.3
*LMOf2365_1515*	Transcription elongation factor GreA	3.4
*nusG LMOf2365_0258*	Transcription antitermination factor NusG	3.4
*LMOf2365_2467*	Phosphate transport system protein PhoU	3.4
*LMOf2365_2223*	MecA family protein	3.6
*LMOf2365_0023*	Transcriptional regulator, GntR family	3.6
*LMOf2365_0576*	Putative DNA-binding transcriptional regulator	3.6
*LMOf2365_2337*	Transcriptional regulator, DeoR family	3.7
*ctsR LMOf2365_0241*	Transcriptional regulator CtsR	3.8
*LMOf2365_0119*	Transcriptional regulator, ArsR family	4.0
*LMOf2365_0446*	Transcriptional regulator, LysR family	4.0
*LMOf2365_2017*	Transcriptional regulator, LacI family	4.1
*LMOf2365_2841*	Transcriptional regulator, AraC family	4.4
*LMOf2365_1051*	Transcriptional regulator, LacI family	4.4
*LMOf2365_0906*	Conserved hypothetical protein	4.8
*LMOf2365_0794*	ROK family protein	5.1
*LMOf2365_2466*	Transcriptional regulator, ArsR family	5.8
*LMOf2365_2669*	Transcriptional regulator, TetR family	5.8
*LMOf2365_0266*	Transcriptional regulator, DegA family	6.1
*LMOf2365_0665*	Rrf2 family protein	6.5
*LMOf2365_0841*	Transcriptional regulator, MerR family	7.7
*LMOf2365_0394*	Transcriptional regulator, DeoR family	9.5
*LMOf2365_1894*	DeoR family transcriptional regulator, catabolite repression regulator	11.5
*LMOf2365_2224*	ArsC family protein	11.7
*LMOf2365_0940*	PRD/PTS system IIA 2 domain protein	12.4
*LMOf2365_2322*	LysR family transcriptional regulator, regulator of the ytmI operon	13.1
*LMOf2365_0435*	DNA-binding protein	14.2
*LMOf2365_2799*	DNA-binding protein	14.7
**Gene ID**	**Gene product**	**Transcript levels**
*LMOf2365_1010*	Transcriptional regulator, MarR family	18.4
*LMOf2365_2233*	Transcriptional regulator, MarR family	19.1
*LMOf2365_0755*	Transcriptional regulator, PadR family	19.5
*LMOf2365_0387*	GntR family transcriptional regulator	25.7
*LMOf2365_0326*	DNA-binding protein	41.2

**TABLE 9:** Transcript levels decreased for select genes in response to bile in anaerobic conditions at pH 5.5.

**Gene ID**	**Gene name**	**Transcript levels**
***Metabolism***		
*LMOf2365_2610*	Putative lipoprotein	−29.9
*LMOf2365_0802*	FMN-dependent NADH-azoreductase	−21.6
*LMOf2365_1226*	Putative peptidase	−18.2
*LMOf2365_0565*	6-phospho-beta-glucosidase	−18.2
*pflA LMOf2365_1426*	Pyruvate formate lyase activating enzyme	−11.1
*LMOf2365_1975*	Riboflavin transporter	−10.2
*pyrH LMOf2365_1330*	Uridylate kinase	−8.7
*LMOf2365_1597*	Bifunctional oligoribonuclease and PAP phosphatase NrnA	−8.5
*LMOf2365_0277*	Glycosyl hydrolase, family 1	−8.5
*LMOf2365_0776*	Hydrolase, alpha/beta fold family	−8.2
*pfl-2 LMOf2365_1946*	Formate C-acetyltransferase	−8.2
*rplS LMOf2365_1814*	Large subunit ribosomal protein L19	−7.7
*pepQ LMOf2365_1600*	Proline dipeptidase	−7.6
*cadA LMOf2365_0672*	Zn2+/Cd2+-exporting ATPase	−7.6
*LMOf2365_2666*	Cell division protein, FtsW/RodA/SpoVE family	−7.3
*LMOf2365_0021*	Glycosyl hydrolase, family 1	−6.9
*LMOf2365_2146*	Hydrogen peroxide-dependent heme synthase	−6.5
*glmS LMOf2365_0762*	Glutamine—fructose-6-phosphate transaminase	−6.3
*LMOf2365_1093*	N-acetylmuramoyl-L-alanine amidase	−6.3
*LMOf2365_0057*	Accessory gene regulator B	−5.9
*LMOf2365_1386*	Phosphate butyryltransferase	−5.7
*thiI LMOf2365_1614*	tRNA uracil 4-sulfurtransferase	−5.7
*galU LMOf2365_1099*	UTP–glucose-1-phosphate uridylyltransferase	−5.6
*LMOf2365_1702*	Methionine synthase/methylenetetrahydrofolate reductase (NADPH)	−5.6
*LMOf2365_2609*	FAD:protein FMN transferase	−5.6
*eno LMOf2365_2428*	Enolase	−5.5
*LMOf2365_2670*	N-acetylmuramoyl-L-alanine amidase, family 4	−5.3
*fabI LMOf2365_0990*	Enoyl-[acyl-carrier-protein] reductase I	−5.2
*LMOf2365_1880*	Copper chaperone; heavy metal binding protein	−5.1
*LMOf2365_2711*	PhnB protein	−5.1
*LMOf2365_2673*	Orn/Lys/Arg decarboxylase	−5.1
*LMOf2365_1368*	Rhodanese-like domain protein	−5.0
*LMOf2365_2510*	UDP-N-acetylglucosamine 2-epimerase	−4.8
*mraY LMOf2365_2069*	Phospho-N-acetylmuramoyl-pentapeptide-transferase	−4.7
*purA LMOf2365_0065*	Adenylosuccinate synthase	−4.7
*ald LMOf2365_1601*	Alanine dehydrogenase	−4.7
*plcA LMOf2365_0212*	1-phosphatidylinositol phosphodiesterase	−4.6
*menE LMOf2365_1696*	O-succinylbenzoate–CoA ligase	−4.6
*murC LMOf2365_1627*	UDP-N-acetylmuramate–alanine ligase	−4.5
*LMOf2365_2743*	Hydrolase, CocE/NonD family	−4.4
*gpmA LMOf2365_2429*	2,3-bisphosphoglycerate-independent phosphoglycerate mutase	−4.4
*LMOf2365_0434*	Peptidoglycan-N-acetylglucosamine deacetylase	−4.1
*tmk LMOf2365_2672*	Thymidylate kinase	−4.1
*LMOf2365_1643*	8-oxo-dGTP diphosphatase	−4.1
*LMOf2365_2133*	Pyridoxal 5′-phosphate synthase pdxS subunit	−3.9
*pyk LMOf2365_1592*	Pyruvate kinase	−3.9
*alaS LMOf2365_1523*	Alanyl-tRNA synthetase	−3.9
*fhs LMOf2365_1906*	Formate–tetrahydrofolate ligase	−3.9
*LMOf2365_1033*	N-acetyldiaminopimelate deacetylase	−3.8
*LMOf2365_0872*	D-alanine-D-alanine ligase	−3.8
*LMOf2365_0987*	Putative GTP pyrophosphokinase	−3.8
*LMOf2365_1299*	Acyl-CoA thioester hydrolase	−3.8
*LMOf2365_1512*	Peptidase, M3 family	−3.7
*pfl-1 LMOf2365_1425*	Formate C-acetyltransferase	−3.7
*LMOf2365_2144*	Nitroreductase family protein	−3.6
*folA LMOf2365_1903*	Dihydrofolate reductase	−3.6
*LMOf2365_1371*	Xaa-Pro aminopeptidase	−3.6
*upp LMOf2365_2511*	Uracil phosphoribosyltransferase	−3.5
*uppS LMOf2365_133*	Undecaprenyl diphosphate synthase	−3.5
*LMOf2365_0239*	Dihydrouridine synthase family protein	−3.5
*LMOf2365_1633*	Putative glutamyl-aminopeptidase	−3.4
*LMOf2365_1476*	[pyruvate, water dikinase]-phosphate phosphotransferase	−3.4
*LMOf2365_0293*	Acetyltransferase, GNAT family	−3.4
*LMOf2365_1691*	L-lactate dehydrogenase	−3.3
*LMOf2365_0101*	Oxidoreductase, aldo/keto reductase family	−−3.3
*LMOf2365_1644*	ADP-dependent NAD(P)H-hydrate dehydratase	−3.3
*LMOf2365_0846*	Pyruvate-ferredoxin/flavodoxin oxidoreductase	−3.3
*LMOf2365_1915*	Carboxypeptidase Taq	−3.3
*hemE LMOf2365_2245*	Uroporphyrinogen decarboxylase	−3.3
*nrdD LMOf2365_0299*	Ribonucleoside-triphosphate reductase	−3.3
*sdhB LMOf2365_1841*	L-serine dehydratase	−3.3
*LMOf2365_2207*	Oxidoreductase, short-chain dehydrogenase/reductase family	−3.2
*LMOf2365_2514*	L-threonylcarbamoyladenylate synthase	−3.2
*pepT LMOf2365_1805*	Tripeptide aminopeptidase	−3.1
*LMOf2365_1048*	Ribonuclease J	−3.1
*mpl LMOf2365_0214*	Zinc metalloproteinase	−3.1
*LMOf2365_0488*	Undecaprenyl diphosphate synthase	−3.1
*LMOf2365_2308*	Bleomycin hydrolase	−3.1
*manA LMOf2365_2143*	Mannose-6-phosphate isomerase, class I	−3.0
*ftsX LMOf2365_2479*	Cell division ABC transporter, permease protein FtsX	−3.0
*gap LMOf2365_2432*	Glyceraldehyde 3-phosphate dehydrogenase	−3.0
***Pathogenesis***		
*plcB LMOf2365_0216*	Phospholipase C	−10.0
*LMOf2365_1812*	Internalin family protein	−6.1
***Replication and repair***		
*dnaE LMOf2365_1596*	DNA polymerase III subunit alpha	−4.9
*LMOf2365_1628*	DNA segregation ATPase FtsK/SpoIIIE, S-DNA-T family	−4.3
*ligA LMOf2365_1783*	DNA ligase, NAD-dependent	−3.2
*recG LMOf2365_1839*	ATP-dependent DNA helicase RecG	−3.0
***Transcription factor***		
*LMOf2365_2335*	Transcriptional regulator, RofA family	−8.6
*argR LMOf2365_1384*	Arginine repressor	−4.4
*LMOf2365_2715*	Transcriptional regulator, MerR family	−3.4
*LMOf2365_2780*	DNA-binding protein	−3.2
***Membrane transport***		
*LMOf2365_2388*	D-methionine transport system substrate-binding protein	−9.1
*LMOf2365_0606*	Putative membrane protein	−8.4
*Ffh LMOf2365_1828*	Signal recognition particle subunit SRP54	−7.3
*LMOf2365_2553*	Putative ABC transport system permease protein	−6.3
*ptsI LMOf2365_1024*	Phosphoenolpyruvate-protein phosphotransferase	−5.9
*LMOf2365_0803*	D-serine/D-alanine/glycine transporter	−5.8
*agrC LMOf2365_0059*	Two-component system, LytTR family, sensor histidine kinase AgrC	−5.1
*LMOf2365_0673*	Putative membrane protein	−4.4
*cydD LMOf2365_2695*	ATP-binding cassette, subfamily C, bacterial CydC	−4.3
*LMOf2365_1034*	Moderate conductance mechanosensitive channel	−4.3
*prf1 LMOf2365_2516*	Peptide chain release factor 1	−4.2
*ldh-1 LMOf2365_0221*	L-lactate dehydrogenase	−4.2
*LMOf2365_2148*	ABC transporter, permease protein	−4.0
*LMOf2365_1450*	ABC transporter, ATP-binding protein	−3.8
*LMOf2365_1994*	ABC-2 type transport system ATP-binding protein	−3.8
*LMOf2365_1264*	Putative transporter	−3.3
*LMOf2365_2323*	Monovalent cation/hydrogen antiporter	−3.2
*LMOf2365_0845*	Na/Pi-cotransporter family protein	−3.2
*LMOf2365_1091*	Teichoic acid transport system permease protein	−3.1
*LMOf2365_2844*	YidC/Oxa1 family membrane protein insertase	−3.0
*LMOf2365_0317*	Putative membrane protein	−3.0
***Translation***		
*tsf LMOf2365_1678*	Elongation factor Ts	−11.4
*rpsB LMOf2365_1679*	Small subunit ribosomal protein S2	−5.4
*valS LMOf2365_1573*	Valyl-tRNA synthetase	−4.1
*gatB MOf2365_1779*	Aspartyl-tRNA(Asn)/glutamyl-tRNA(Gln) amidotransferase subunit B	−4.1
*efp LMOf2365_1372*	Translation elongation factor P	−3.4
*thrS LMOf2365_1580*	Threonyl-tRNA synthetase	−3.1
*infA LMOf2365_2583*	Translation initiation factor IF-1	−3.1

## Discussion

### Anaerobiosis Improves Survival of *L. monocytogenes* in Conditions Mimicking the Gastrointestinal Tract

Survival of *L. monocytogenes* strain F2365 was analyzed under conditions mimicking the GI tract. This strain was chosen as it is a serotype 4b strain, which represents the serotype of a large portion of outbreak strains. F2365 was isolated from one of the deadliest outbreaks of *L. monocytogenes* (Linnan et al., 1988). F2365 has been sequenced (Nelson et al., 2004) and has been extensively studied for genomic analyses (Chatterjee et al., 2006; Liu and Ream, 2008; Payne et al., 2013), making it an ideal strain to analyze transcriptomic responses.

Bile is made in the liver, stored in the gall bladder, and released to the duodenum upon ingestion. The environment in the gall bladder is anaerobic and neutral pH, while the duodenum is acidic and microaerophilic (Zheng et al., 2015). The alterations in oxygen availability within the GI tract are essential to developing the redox relationship between microbes and host (He et al., 1999; Espey, 2013). Therefore, we tested how oxygen influenced the survival of *L. monocytogenes* under either acidic (mimicking the duodenum) or neutral (mimicking the gall bladder) bile conditions.

Since variations in transcript levels were observed due to alterations in oxygen availability, we wanted to determine which genes were commonly expressed under anaerobiosis. Transcript levels of five genes were found to be up-regulated under exposure to anaerobic conditions regardless of whether the cultivation was conducted under either neutral or acidic pH (Table 2), though there were differential expressions between the two conditions. Transcripts common to both conditions included two membrane transporters *LMOf2365_2333* and *cadA* (*LMOf2365_0672*), two chaperones, and the stress response related gene *gadG* (*LMOf2365_2405*). CadA has been previously shown to be involved in formation of biofilms at 25°C by *L. monocytogenes* (Parsons et al., 2017). CadA also has been implicated in having roles in virulence and pathogenesis (Parsons et al., 2017). Therefore, it is possible that CadA is involved in stress response mechanisms related to anaerobic survival and that the formation of biofilms may be a critical component to survival. Previous studies have also shown that various stressors (i.e., heat shock, nutrient limitation, acidic condition, etc.) cause an increase in the expression of chaperones (Wright et al., 2016). Indeed, the data showed an increase in the transcript levels of two chaperones (*dnaK* and *dnaJ*) under anaerobic conditions at both pH 7.5 and 5.5. Therefore, it is possible that *L. monocytogenes* uses molecular chaperones to combat anaerobic stress, which in turn assists with phagocytosis. The *gadG* encodes for an amino acid antiporter that is part of the glutamate decarboxylase system, which is a defense mechanism up-regulated by *L. monocytogenes* under acid stress and anaerobiosis. This system alleviates the acidification of the cytoplasm by consuming a proton (Cotter et al., 2001; Jydegaard-Axelsen et al., 2004; Paudyal et al., 2020). The fact that this transcript was up-regulated in response to anaerobic conditions suggests that there may be overlapping functions of the GAD system in both acid resistance and anaerobiosis. The transcript level of the *LMOf2365_2333* gene was increased by nearly 9-fold in comparison to acidic pH. There is a possibility that this amino acid anti-transporter may function with *gadG* in response to bile. This should be further explored in future studies.

Transcript levels of eighteen genes were down-regulated under anaerobic conditions regardless of the pH, including histidine kinase, metabolic genes, a universal stress response gene, and genes coding for hypothetical proteins. As histidine kinases are involved in two-component systems, it is possible that suppression of this sensor is responsible for the response to oxygen availability. One of the metabolic genes, the phosphoglycerate mutase, has been shown in *Bacillus subtilis* to be responsible for the control of the two-component system required for sensing and responding to aerobic and anaerobic respiration (Nakano et al., 1999). The fact that the transcript level of this gene was down-regulated suggests that the accumulation of the product 1,3-bisphosphoglycerate, which is the intermediate in the reaction catalyzed by phosphoglycerate mutase, might impact the regulation of the histidine kinase *LMOf2365_2554*. The impact of this precursor on regulation of two-component systems needs to be explored in further detail. The transcript level of the gene *acpP* was also down-regulated. This gene product is involved in biosynthesis of fatty acids as a lipid transporter. This gene has been found to be differentially regulated under anaerobic conditions in many other bacteria, including *Escherichia coli* and *Neisseria gonorrhoeae* (Isabella and Clark, 2011). This indicates that the regulation of the fatty acid synthesis is necessary for the adaptability to anaerobiosis.

### Differential Transcript Levels in Response to Anaerobic Acidic Conditions

An increase in the transcript levels of *nrdD* (*LMOf2365_0299*), which is an anaerobic ribonucleoside-triphosphate reductase that catalyzes the synthesis of dNTPs required for DNA replication, was observed under anaerobic conditions at acidic pH. NrdD is an essential enzyme required by *L. monocytogenes* and other GI pathogens, such as *E. coli*, to survive under anaerobic conditions (Garriga et al., 1996; Ofer et al., 2011). Since our study showed acidic conditions influence the up-regulation of this gene under anaerobic conditions, there is a possibility that this enzyme is involved in growth under acidic conditions. This may be required to stabilize the redox potential of the cell under acidic conditions. Ribonucleotide reductases have been explored as potential biomedical targets for bacterial infections (Torrents, 2014). Since the ribonucleotide reductase was up-regulated under anaerobic acidic conditions, it will be necessary for future studies to analyze the activity of antibacterial compounds under these conditions to effectively target the protein expressed.

Transcript levels of genes coding for a glycosyl hydrolases, which are involved in hydrolyzing the glycosidic linkages in sugars, were also up-regulated. Certain glycosyl hydrolases have been previously identified as virulence factors in gram positive pathogenic bacteria, including *Streptococcus pneumoniae* (Niu et al., 2013). Glycosyl hydrolase PssZ has been observed to degrade extracellular polymeric substance, thereby disrupting biofilm formation by *L. monocytogenes* (Wu et al., 2019). *L. monocytogenes*, which is an intracellular bacterium, may synthesize glycosyl hydrolases upon exposure to acidic pH under anaerobic conditions, which thereby hinders formation of biofilms and facilitates the bacterium’s entry into the host cells.

One of the virulence factors of *L. monocytogenes* is metalloproteases. Few such proteases were identified to have an increase in transcript levels at pH 5.5 in anaerobic conditions, including the aminopeptidase (*LMOf2365_2308*) (Table 4). It has been shown that the bacterial burden of *L. monocytogenes* EGDe strain in host cells decreased significantly when the aminopeptidase T of family M29 was deleted (Cheng et al., 2015). Thus, at anaerobic conditions under acidic pH, aminopeptidases may be up-regulated and function as virulence factors.

GalU (*LMOf2365_1099*), UTP-glucose-1-phosphate uridyltransferase, which catalyzes cell wall teichoic acid glycosylation, had an increase in transcript levels under anaerobic conditions at pH 5.5 (Table 4; Kuenemann et al., 2018). *In silico* design of GalU inhibitors attenuated virulence of *L. monocytogenes*, proving GalU to be an instrumental part in virulence pathways (Kuenemann et al., 2018). Various transcription factors were up-regulated under anaerobic conditions at pH 5.5 (Table 4), including the *fur* regulator that controls virulence of various pathogenic bacteria. We also observed that transcripts coding for virulence genes, such as listeriolysin O and internalin family proteins, were also up-regulated under these conditions. The transcript level of a methyl accepting chemotaxis protein was also increased. In *L. monocytogenes* chemotaxis genes *cheA* and *cheY* have been shown to facilitate to adhesion and thereby invasion into the host epithelial cells. As *L. monocytogenes* is an intracellular pathogen, it may be possible that along with the CheA and CheY system, it is using the methyl accepting chemotaxis proteins to attach to epithelial cells under anaerobic conditions at pH 5.5 (Dons et al., 2004).

Internalins A and B are required by *L. monocytogenes* for facilitating entry inside host cells. Transcript levels for genes encoding internalin proteins were found to be up-regulated under the acidic environment in absence of oxygen. Interestingly, the transcript level of *inlE* (*LMOf2365_0283*), which is a gene coding for the secreted protein Internalin E, was decreased. Internalins A and B are involved in adhesion and invasion by *Listeria*, but Internalin E is not involved in invasion (Dramsi et al., 1997). This indicates anaerobiosis influences the invasive potential of *L. monocytogenes*. The impact of anaerobiosis on invasion has been shown *in vitro* and *in vivo*, but the exact mechanism of such interplay has not been well characterized (Bo Andersen et al., 2007; Harris et al., 2019).

### Differential Transcript Levels in Response to Bile Under Anaerobic Conditions

Previous studies have shown that following ingestion of *L. monocytogenes* into host systems, the *prfA* regulon is up-regulated (Scortti et al., 2007). *prfA*, the positive regulatory factor A, is a transcription factor that regulates major virulence factors of *L. monocytogenes*. *prfA* regulates listeriolysin O, phospholipase C and metalloproteases, all of which were up-regulated in anaerobiosis in presence of bile (Table 6). Following bile exposure, the transcript levels of the virulence regulator *prfA* were decreased (Boonmee et al., 2019); however these data show that under anaerobic conditions in presence of bile, *prfA* is up-regulated independent of pH. We have also observed that *L. monocytogenes* survives bile better under anaerobic conditions (Figure 2).

Previous transcriptomics studies in *L. monocytogenes* 10403S (Boonmee et al., 2019) have found that following exposure to bile, the house keeping sigma factor σ^A^ has a significant role in survival. *marR* [multiple antibiotic resistance regulator (*LMOf2365_0641*)] is a transcriptional regulator that was up-regulated in response to bile in anaerobic conditions regardless of the pH tested (Table 6). In pathogens such as *Salmonella* and *Staphylococcus*, *marR* homologs *slyA* and *sarZ* regulate virulence gene expression. *marR* homologs have also been found to regulate genes involved in stress response, degradation or efflux of harmful substances and metabolic pathways (Grove, 2013). Bile exposure under anaerobic environments may trigger the up-regulation of *marR* to export bile out of the bacterial cell, thereby contributing to the bile resistance of *L. monocytogenes* along with other factors. The role of *marR* in bile resistance needs to be further explored.

Glutamine synthetase catalyzes the condensation of ammonia and glutamate to form glutamine. The transcript level of the glutamine synthetase repressor, *glnR* (*LMOf2365_1316*) was increased following exposure to bile in anaerobic conditions. It is a central nitrogen metabolism regulator which is activated in presence of glutamine. When glutamine is in excess, GlnR represses the synthesis of glutamine synthetase (Kaspar et al., 2014). Another probable transcriptional regulator (*tnrA* or *codY*) represses glutamine synthetase and its activation have been found to be essential in replication *Listeria* intracellularly (Kaspar et al., 2014). Interestingly glutamine synthetase was also up-regulated under the same conditions, which indicates the possibility of a feedback loop.

Metalloenzyme carbonic acid catalyzes hydration of carbon dioxide into bicarbonate and proton (Supuran, 2016). The infection cycle of *Legionella* has similarities with that of *L. monocytogenes*, such as invasion and escaping the phagosome. *Legionella* has been shown to evade the destruction by maintaining neutral pH (Supuran, 2016). One of the enzymes involved in regulating the pH is carbonic anhydrase; the transcript level of carbonic anhydrase increased under anaerobic conditions in the presence of bile in *L. monocytogenes* (Table 6). This could indicate that environmental conditions mimicking parts of intestine can contribute to *Listeria’s* pathogenic potential. Interestingly, the transcript level of this gene was down-regulated under acidic conditions (Table 5), suggesting that the influence of bile is important to the expression of this gene.

Transcript levels representative of an uncharacterized membrane protein *LMOf2365_1012* that belongs to the TerC family was up-regulated following exposure to bile in anaerobic conditions (Table 6). In *B. subtilis*, TerC has been found to confer manganese resistance (Paruthiyil et al., 2020). In *Streptococcus*, manganese homeostasis is linked to oxidative stress as well as virulence (Turner et al., 2015). It is possible that TerC is linked with manganese homeostasis and therefore virulence in the presence of bile under anaerobic conditions. Transcripts coding for several other membrane transporters were also increased in their levels under the anaerobic environment in response to bile. The zinc ABC transporter has been shown to have a role in virulence of *L. monocytogenes* in a mouse infection model (Corbett et al., 2012). Thus, bile exposure in absence of oxygen probably impacts uptake of zinc by the bacteria thereby impacting the virulence. NRAMP, which functions as a metal ion transporter on membranes, was up-regulated (Nevo and Nelson, 2006).

The transcript level of the oligopeptide ABC transporter, which is an oligopeptide binding protein that helps the bacteria survive intracellularly, was increased (Slamti and Lereclus, 2019). It is the substrate binding component or receptor of an ABC type oligopeptide transport system that binds extracellular peptides, relays it to the membrane component of the system and inside the bacterial cell afterward. Gram positive bacteria such as *Listeria*, *Streptococcus*, and *Enterococcus*, use peptides to sense and respond to environmental changes. The gene *oppA*, which encodes for an oligopeptide binding protein, has been found to be required for invasion (Borezee et al., 2000). Thus, the oligopeptide ABC transporter observed in our study could be responsible for intracellular survival of bacteria in presence of bile under anaerobic conditions.

Interestingly, there was a decrease in the transcript levels of *hly* (*LMOf2365_0213*), which encodes for listeriolysin O, at both pH 7.5 and 5.5 following exposure to bile under anaerobic conditions. This was different than what was observed under anaerobiosis at pH 5.5 alone, as *hly* (LMOf2365_0213) was up-regulated in these conditions (Table 4). This suggests that bile has an important role in regulating the invasiveness of *L. monocytogenes*. This correlates well with previous studies that have shown that *L. monocytogenes* remains extracellular in the gall bladder, which has high concentrations of bile (Hardy et al., 2004; Dowd et al., 2011).

### Differential Transcript Levels in Response to Bile Under Acidic and Anaerobic Conditions

There was an increase in transcript levels for the myosin cross reactive antigen (McrA) (*LMOF2365_0508*; Table 8). Although its function in *L. monocytogenes* is yet unknown, in *Streptococcus pyogenes* McrA is a fatty acid double bond hydratase that adds water to double bonds of fatty acids. Upon deletion of this gene, decreased oleic acid resistance and reduced adherence and internalization in the host cell was observed in *S. pyogenes* (Volkov et al., 2010). Conditions encountered within the duodenum may directly or indirectly contribute to up-regulation of *mcrA*, which may regulate the pathogen’s resistance to bile.

Internalin E and hemolysin III are both virulence factors responsible for internalization and invasion for *L. monocytogenes*. Both had an increase in transcript levels, indicating that bile exposure at acidic and anaerobic conditions, which mimics the duodenum, is conducive to the pathogenesis of the bacteria.

The transcript level of the LPXTG-motif cell wall anchor domain (*LMOF2365_1144*) was also up-regulated. In the *L. monocytogenes* EGDe strain, it has been shown that a LPXTG protein encoded by the *Listeria* mucin binding invasion A gene, or *lmiA*, has roles in promoting bacterial adhesion and entry into the host cell (Mariscotti et al., 2014). MucBP domain present in LPXTG was observed to bind to mucin. Thus, up-regulation of LPXTG gene under conditions mimicking the duodenum indicates that these conditions may facilitate invasion of host cells by the bacteria.

The level of transcripts representing flagellin also increased. It has been shown that flagellin helps in motility soon after ingestion *in vivo* (O’Neil and Marquis, 2006) and invasion (Dons et al., 2004). A previous study has also observed up-regulation of motility under exposure to bile at pH 5.5 (Guariglia-Oropeza et al., 2018). The fact that expression increased in conditions that would be encountered soon after ingestion suggests that the flagellin are important for the motility of the bacteria to the location in the GI tract where they will invade the intestinal lining.

The transcript level of the histidine kinase *LMOf2365_2554* was also up-regulated under conditions mimicking the duodenum. Histidine kinase is the signal receiver a two-component regulatory system. Its counterpart in the system is the response regulator (Chang and Stewart, 1998; Stock et al., 2000; West and Stock, 2001; Krell et al., 2010). Response regulators in *L. monocytogenes* have been proven to have roles in virulence and pathogenesis. Sensor histidine kinase, ChiS, regulates the chitin utilization pathway required by *Vibrio cholerae*, which is needed to survive in aquatic environments. Chourashi et al. (2016) observed that ChiS has an important role in adherence and intracellular survival of *V. cholerae* in HT-29 cell cultures. They also showed that the sensor histidine kinase ChiS was activated in the presence of intestinal mucin (Chourashi et al., 2016). In the case of *L. monocytogenes*, it could be possible that the conditions in the duodenum are favorable for activation of the sensor histidine kinase, which could in turn relay information that would result in the activation of transcription factors responsible for adhesion and invasion.

Transcript levels representative of replication and repair genes were also up-regulated. In *L. monocytogenes* strain EGDe, RecA has been shown to have roles in bile and acid resistance, as well as in adhesion and invasion to Caco-2 cell cultures (van der Veen and Abee, 2011). Our data indicate that in the pathogenic strain F2365, RecA has the similar role of bile and acid resistance. In our study, we have also found that under anaerobic conditions (along with bile and acidic) the transcript level of *recA* changed, indicating absence of oxygen may have impact on activation of RecA.

The transcript level for a gene encoding for the transcriptional regulator *padR* was up-regulated (Table 8). In *L. monocytogenes* EGDe, LftR, which is a PadR like transcriptional regulator, has been shown to influence invasion of human host cells (Kaval et al., 2015). It is already known that *Listeria* uses internalin proteins for adhering and internalizing into the cell. Kaval et al. (2015) found that LftR, which is an uncharacterized protein, is required for invasion.

Transcript level of the gene encoding for *ctsR*, (LMOf2365_0241) a class III stress gene repressor that negatively regulates *clp*, was up-regulated under these conditions (Table 8). CtsR has been shown to be required for virulence in mice. PrfA which regulates many virulence genes of *L. monocytogenes* has been shown to down-regulate ClpC production (Karatzas et al., 2003). Although Karatzas et al. (2003) could not find any relationship between *clp* and *prfA*, there is still a possibility that there is a connection between the regulation of Clp by CtsR under anaerobic conditions in exposure to bile at acidic pH (Cui et al., 2018).

The transcript level of the transcription elongation factor *greA* (*LMOf2365_1515*) also increased under anaerobic conditions with acidic bile. GreA has been found to have roles in affecting functions of virulence gene expression in the pathogen *Francisella tularensis* subsp. Novicida (Cui et al., 2018). In *F. tularensis*, GreA was found to be required for invasion and intracellular growth of bacteria. Cui et al. (2018) also observed suppression of virulence of the *greA* mutant in mouse model. Transcriptomics analysis of the *greA* mutant revealed down-regulation of various genes responsible for virulence. Thus, with respect to our work, conditions in the duodenum are favorable for induction of the transcription elongation factor *greA*, which may in turn regulate genes responsible for invasion and multiplication of *L. monocytogenes*.

This study indicates that not only one stressor, but combinations of different stressors impact the transcription of various virulence genes. Transcriptomic and phenotypic studies in absence of these genes under mimicking physiological condition could give us an insight into this mechanism. A better understanding of how these biological processes help the survival of *L. monocytogenes* will lead us to understand how the physiological conditions contribute to the pathogenesis.

## Materials and Methods

### Bacterial Strain and Culture Conditions

*Listeria monocytogenes* str. 4b F2365 was used for this study. Overnight cultures of *L. monocytogenes* str. 4b F2365 were grown at 37°C aerobically in Brain Heart Infusion (BHI) media at pH 7.5. Next day, inoculum (1:100) from the overnight culture was used to grow the cells to mid exponential phase in fresh BHI media (OD_600_ = 0.3 to 0.5) under either aerobic or anaerobic conditions in 5 mL aliquots. Anaerobic culture conditions were obtained using an incubator shaker set at 37°C inside a Coy Anaerobic Chamber with a gas mixture of 95% N_2_ and 5% H_2_ (Coy Laboratory Products, United States). Cells were then pelleted at 8000 × *g* at 23°C and resuspended in fresh BHI at a pH of either 7.5 or 5.5; pH was adjusted with either HCl or NaOH. For bile treated cells, mid exponential phase cells were resuspended in BHI at a pH of either 7.5 or 5.5 supplemented with 1% porcine bile extract (Sigma Aldrich, United States). Cells were then grown under either aerobic or anaerobic conditions at 37°C. This study had eight different conditions that mimicked parts of the GI tract. The conditions tested were: (1) aerobic at pH 5.5; (2) anaerobic at pH 5.5; (3) aerobic at pH 7.5; (4) anaerobic at pH 7.5; (5) aerobic at pH 5.5 with 1% porcine bile; (6) anaerobic at pH 5.5 with 1% porcine bile; (7) aerobic at pH 7.5 with 1% porcine bile; and (8) anaerobic at pH 7.5 with 1% porcine bile. For each time point during a 7 h incubation period, aliquots were serially diluted in phosphate buffered saline (PBS) and plated onto BHI agar plates. Plates were incubated overnight at 37°C prior to enumeration. Three independent replicates were performed in parallel for each individual condition tested.

### RNA Extraction, Library Preparation and RNA Sequencing

To isolate the RNA for analysis of the transcript level expression, cells were collected after 1 h of incubation in the eight culture conditions described above. Three biological replicates were assayed. Briefly, 5 mL of culture was pelleted by centrifugation at 8,000 × *g* for 5 min at room temperature. Cell pellets were then treated with RNA Protect Bacterial Reagent (Qiagen, Germany). Total RNA was isolated using the RNeasy^®^ Mini Kit (Qiagen, Germany) per manufacturer’s instructions. The extracted RNA was quantitated using Qubit 3 Fluorometer (Invitrogen, United States) using the Qubit RNA BR assay kit (Thermo Fisher, United States). Extracted samples with values of A260/280 ∼ 2.0 were selected for sequencing. Illumina HiSeq^TM^ 2000 paired-end 50 bp sequencer (PE50) was used. Ribosomal RNA was reduced with Epicentre RiboMinus kit (Illumina, United States) coupled with Directional RNA-Seq library prep with TruSeq indexes (Illumina, United States) per manufacturer’s instructions.

### Data Analysis

Differences in survival were determined using a student’s *t*-test (Prism 8). Tophat-2.0.8.b (Trapnell et al., 2009) was used to align the RNA-Seq data to the reference genome, AE017262.2 *L. monocytogenes* str. 4b F2365. Transcript level calculation and FPKM normalization were performed using Cufflinks-2.1.1 (Trapnell et al., 2010). FPKM filtering cutoff of 1.0 was maintained to determine expressed transcripts. Differential transcript levels of the genes were determined using Cuffdiff (Trapnell et al., 2013). Differential transcript levels which had a greater than 3-fold expression and were statistically significant (*p* < 0.01 and *q* < 0.01) were subjected to Gene Ontology (GO) enrichment analysis using Blast2GO (Conesa et al., 2005). In this software, the up- and down-regulated transcripts were selected, and BLAST was performed against the *L. monocytogenes* nucleotide database in NCBI. The BLAST results were then mapped and annotated.

## Data Availability Statement

SRA IDs of the submitted data: SRR13859772, SRR13859774, and SRR13859773: F2365 pH 5.5 Aerobic, SRR13859144, SRR13859143, and SRR13859142: F2365 pH 5.5 Anaerobic, SRR13859527, SRR13859526, and SRR13859525: F2365 pH 5.5+ Bile Anaerobic, SRR13859600, SRR13859599, and SRR13859598: F2365 pH 7.5+ Bile aerobic, SRR13858938, SRR13858937, and SRR13858936: F2365 pH 7.5+ Bile Anaerobic, SRR13858765, SRR13858767, and SRR13858766: F2365 pH 7.5 Anaerobic, SRR13853432, SRR13853433, and SRR13853431: F2365 pH 5.5+ Bile Aerobic, SRR13849951, SRR13849952, and SRR13849950: F2365 pH 7.5 aerobic.

## Author Contributions

JD: conceptualization, supervision, and project administration. MA, MD, JD, GS, and DC: methodology. GS and DC: software. GS, DC, and JD: validation and visualization. DC and JD: investigation and writing–review and editing. MA and GS: resources. DC: data curation and writing–original draft preparation. All authors have read and agreed to the published version of the manuscript.

## Conflict of Interest

The authors declare that the research was conducted in the absence of any commercial or financial relationships that could be construed as a potential conflict of interest.

## Publisher’s Note

All claims expressed in this article are solely those of the authors and do not necessarily represent those of their affiliated organizations, or those of the publisher, the editors and the reviewers. Any product that may be evaluated in this article, or claim that may be made by its manufacturer, is not guaranteed or endorsed by the publisher.
