# Supplementary material for: Transcriptomic Analysis of Listeria monocytogenes in Response to Bile Under Aerobic and Anaerobic Conditions
Source: Front Microbiol. 2021 Nov 11;12:754748. doi: 10.3389/fmicb.2021.754748 (PMC8636025; doi:10.3389/fmicb.2021.754748)
Supplement: Supplementary file 1 [file Data_Sheet_1.DOCX]

5 genes

Figure S1: Comparison of upregulated genes under pH 7.5 & 5.5 aerobic vs anaerobic conditions.

18 genes

Figure S2: Comparison of downregulated genes under pH 7.5 & 5.5 aerobic vs anaerobic conditions.

53 genes

Figure S3: Comparison of upregulated genes under pH 7.5 & 5.5 anaerobic vs bile anaerobic conditions.

9 genes

Figure S4: Comparison of downregulated genes under pH 7.5 & 5.5 anaerobic vs bile anaerobic conditions.
